# Predominance of Dengue Virus Serotype-1/Genotype-I in Eastern and Southeastern Ethiopia

**DOI:** 10.3390/v16081334

**Published:** 2024-08-21

**Authors:** Mesfin Mengesha Tsegaye, Adamu Tayachew Mekonnen, Daniel Tsega Gebretsion, Tesfaye Gelanew, Dawit Hailu Alemayehu, Dessalegn Abeje Tefera, Tamirayehu Seyoum Woldemichael, Bethlehem Adnew Getaneh, Eleni Kidane Abera, Gadissa Gutema Jebessa, Asefa Konde Korkiso, Mengistu Biru Tessema, Admikew Agunie Asfaw, Yoseph Asrat Temre, Mesfin Wossen, Anne Piantadosi, Huachen Zhu, Alemseged Abdissa, Adane Mihret, Andargachew Mulu

**Affiliations:** 1 Viral Diseases Research Division, Armauer Hansen Research Institute, Addis Ababa P.O. Box 1005, Ethiopia; tesfaye.gelanew@ahri.gov.et (T.G.); dawit.hailu@ahri.gov.et (D.H.A.); dessalegn423@gmail.com (D.A.T.); seyoumtam27@gmail.com (T.S.W.); bethlehem.adnew@ahri.gov.et (B.A.G.); alemseged.abdissa@ahri.gov.et (A.A.); adane.mihret@ahri.gov.et (A.M.); andargachew.mulu@ahri.gov.et (A.M.); 2 State Key Laboratory of Emerging Infectious Diseases, School of Public Health, The University of Hong Kong, Hong Kong SAR 999077, China; zhuhch@hku.hk; 3 Public Health Emergency Management Center, Ethiopian Public Health Institute, Addis Ababa P.O. Box 1242, Ethiopia; adamutayachew@gmail.com (A.T.M.); danitsega03@gmail.com (D.T.G.); elikid2003@gmail.com (E.K.A.); gadissagutema@gmail.com (G.G.J.); asefak2010@gmail.com (A.K.K.); MengistuBiru29@gmail.com (M.B.T.); adime12ag21@gmail.com (A.A.A.); yosef.asrat34@gmail.com (Y.A.T.); mesfin.wossen@ephi.gov.et (M.W.); 4 Department of Pathology and Laboratory Medicine, Emory University School of Medicine, Atlanta, GA 30322, USA; anne.piantadosi@emory.edu

**Keywords:** Ethiopia, dengue virus, outbreak, sequence, capsid/pre-membrane (CprM), serotype, genotype, phylogenetic analysis

## Abstract

We determined the dengue virus (DENV) serotypes and genotypes in archived serum samples that were collected during the 2014–2016 and 2021 dengue outbreaks in Dire Dawa City and the Somali region in Ethiopia. DENV serotype 1 (DENV-1) was predominant followed by DENV serotype 2 (DENV-2). Thirteen of the DENV-1 strains were assigned to Genotype-I, while the remaining two were found to be Genotype-III. All three DENV-2 strains were assigned the Cosmopolitan Genotype. The DENV strains responsible for the outbreaks are genetically closely related to the DENV strains that circulated in neighboring and Asian countries. The findings also showed continued local transmission of a monophyletic lineage and a co-circulation of DENV-1 and DENV-2 during the outbreaks. There is a need to strengthen DENV genomic surveillance capacity for the early detection of circulating serotypes, and prevent devastating consequences of future outbreaks due to the co-circulation of different serotypes.

## 1. Introduction

Dengue virus (DENV), the causative agent of dengue fever, is an arthropod-borne virus belonging to the genus *Flavivirus* of the family *Flaviviridae*. DENV is an enveloped, single-stranded and positive-sense RNA virus, with a genome size of about 11 kb and a single open reading frame (ORF). There are four distinct serotypes (DENV 1–4) that are genetically related, with a sequence homology of 65–70%, but are antigenically different [1]. Each DENV serotype can be further categorized into phylogenetically distinct clusters called genotypes which vary in their geographical distributions, epidemic potential, fitness, and virulence [2]. DENV is transmitted to humans mainly by *Aedes aegypti* mosquitoes, but other species within the genus *Aedes* can also transmit the virus [3]. The global incidence of dengue has grown dramatically, with about half of the world’s population now at risk and an estimated 100–400 million infections occurring each year, although over 80% are generally mild and asymptomatic [3]. A meta-analysis of the papers published between 2010 and 2020 in sub-Saharan Africa estimated a high prevalence of DENV infection, with an estimated pooled IgG prevalence of 25%, a pooled IgM prevalence of 10%, and a pooled DENV RNA prevalence of 14% [4].

The first laboratory-confirmed massive outbreak of DENV infection in Ethiopia occurred in Dire Dawa City, Eastern Ethiopia in 2014 [5]. Prior to this outbreak, dengue was reported in travelers/expatriates returning from Ethiopia to Europe and in a seroprevalence study [6]. Since 2014, there have been recurrent outbreaks of dengue fever in Dire Dawa City, as well as the Somali and Afar Regions [5,7,8,9,10,11].

Beyond the essential role of detecting and confirming outbreaks for an epidemic response, Ethiopia lacks the comprehensive molecular characterization of DENV strains. Limited information on circulating serotypes and an absence of data on DENV genotypes pose challenges given the heightened risk of severe dengue with secondary infections of heterologous DENV serotypes, particularly serotypes 2, 3, and 4 [12].

To address this gap and better prepare for future DENV infections and outbreaks, our focus is on determining both the serotypes and genotypes of DENV strains responsible for the documented outbreaks. Our study targeted the capsid/premembrane (CprM) junction region of DENV, recognized for its utility in serotyping and genotyping [13,14,15]. This effort is pivotal in advancing our understanding of the molecular landscape of DENV in Ethiopia, and enhancing our preparedness and response capabilities.

## 2. Methods

### 2.1. Source of Samples

**Figure 1:**
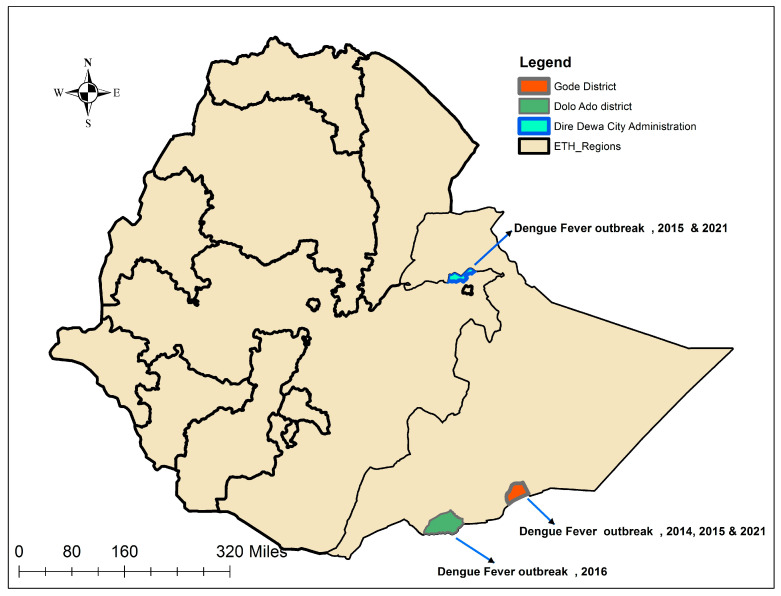
Map of Ethiopia showing districts affected by dengue during in the 2014–2016 and 2021 outbreaks, and from which DENV strains were sequenced.

Archived DENV RNA positive serum samples from the 2014, 2015, 2016, and 2021 dengue outbreaks in Dire Dawa City and Somali Region in Eastern and Southeastern Ethiopia (Figure 1) were obtained from the Ethiopian Public Health Institute (EPHI). The CDC Trioplex Real-Time RT-PCR Assay was used for the detection of DENV RNA [https://www.cdc.gov/zika/pdfs/trioplex-real-time-rt-pcr-assay-instructions-for-use.pdf] (accessed on 5 January 2014).

### 2.2. Dengue RNA Amplification

Viral RNA was extracted from archived serum samples using the QIAamp Viral RNA Mini kit (QIAGEN, Hilden, Germany). Complementary DNA (cDNA) was synthesized using the superscript IV Reverse Transcriptase enzyme (Invitrogen, Carlsbad, CA, USA)) with a DENV-specific reverse primer (5′-GCGCCTTCNGNNGACATCCA-3′, target genome position 764–783 of DENV-1). A nested PCR was performed to amplify the CprM junction. The first round of PCR amplified a 652 bp product using the following forward primer: 5′-TCAATATGCTGAAACGCGCGAGAAACCG-3′ and reverse primer: 5′-GCGCCTTCNGNNGACATCCA-3′, targeting positions 132–153 and 764–783 of the DENV-1, respectively, as described by Alm et al. [16]. For the second round PCR, a 511 bp fragment was amplified using the primer pairs of 5′-TCAATATGCTGAAACGCGCGAGAAACCG-3′ and 5′-TTGCACCAACAGTCAATGTCTTCAGGTTC-3′, as reported previously [17], targeting positions of 132–159 and 614–642 of DENV-1. Primer positions are based on the NCBI reference sequence (GenBank acc. no. NC_001477.1). PCR reactions were run based on the following cycling conditions for the first round of PCR: initial denaturation at 95 °C for 2 min, and 50 cycles of denaturation at 95 °C for 20 s; annealing at 50 °C for 30 s; extension at 70 °C for 30 s and a final extension at 70 °C for 10 min with a final PCR reaction volume of 50 microliter. For the nested PCR, the cycling conditions were similar, except the annealing temperature was increased to 54 °C, in accordance with the melting temperature of the primers. Post-amplification products were visualized on a 1.5% agarose gel. A band of the expected size was excised from the gel and purified using the Pure Link^®^ Quick Gel Extraction Kit (Invitrogen, Carlsbad, CA, USA), according to the manufacturer’s protocol.

### 2.3. Sequencing

Cleaned PCR products were cycle-sequenced using BDT V3.1 (Big Dye Terminator cycle sequencing kit (Applied Biosystems, Foster City, CA, USA)), using the forward and reverse amplification primers of the nested PCR. The cycle-sequenced product was purified using (QIAGEN DyeEx Spin Kit (QIAGEN, Hilden, Germany)) and dried in a vacuum centrifuge. The purified DNA pellet was re-suspended in 10 μL formamide for 30 min and then loaded to the ABI 3500XL automated DNA sequencer (Applied Biosystems, USA) for capillary sequencing.

Sequences from both the forward and reverse primers were trimmed, edited and assembled, and a consensus sequence was generated using Geneious prime ^®^ v.2022.2.1 (https://www.geneious.com/academic/) (accessed on 6 June 2023). Each successful DENV sequence was uploaded to the GenBank database and the accession numbers were acquired (OR227059–OR227076) (Table).

### 2.4. Serotyping and Genotyping, and Phylogenetic Analysis

A nucleotide BLAST search was performed on NCBI to confirm that all the sequences from this study belong to DENV. Then, serotyping and genotyping of the DENV sequences were carried out using the Dengue Virus Typing Tool (version 3.83) of the Genome Detective web-based software (https://www.genomedetective.com/app/typingtool/dengue) (accessed on 15 June 2023) [18,19] and Flavivirus Genotyping Tool (Version 0.1) developed by the National Institute for Public Health and the Environment of the Netherlands, and Emweb, Belgium (https://www.rivm.nl/mpf/typingtool/flavivirus/) (accessed on 20 June 2023). For serotype-specific phylogenetic analysis of the DENV-1 and DENV-2 sequences from this study, a BLAST search was performed for each serotype in the NCBI database, and all sequences of the dengue viruses with top hit sequences in BLAST were selected. Sequences were aligned using the MAFFT sequence alignment program. Short and poor-quality sequences were removed, and sequences were re-aligned using MEGA (version 11.0.13) before phylogenetic tree construction. Serotype-specific trees were constructed using 2442 sequences for DENV-1 and 1911 sequences for DENV-2 using SYM+R5 and SYM+R4 best-fit substitution models, respectively. Genotype-specific smaller trees were constructed using sequences from clusters containing our sequences. All phylogenetic trees were constructed using the maximum-likelihood phylogenies implemented in IQ-TREE v.1.6.12 [20] with bootstrapping of 1000 times, using the best-fit substitution models. All trees were visualized in FigTree v.1.4.4.4 [21] and edited using Adobe illustrator software v. 28.0.

## 3. Result

### 3.1. PCR Detection and Sequencing

A total of 39 RT-PCR-confirmed DENV serum samples were considered for amplification and subsequent serotyping/genotyping. Twenty-one samples had a detectable RNA and were processed further for sequencing. Eighteen samples yielded a good-quality CprM region sequence.

### 3.2. Serotyping and Genotyping

The Flavivirus Genotyping Tool assigned 15 of the 18 sequences from this study a DENV serotype 1 (DENV-1) and the remaining sequences a DENV serotype 2 (DENV-2) (Table). Thirteen of the 15 DENV-1 strains were found to be Genotype-I, while the rest two were Genotype-III (Table). All three DENV-2 viruses were assigned a Cosmopolitan Genotype (Table 1).

**Figure 2:**
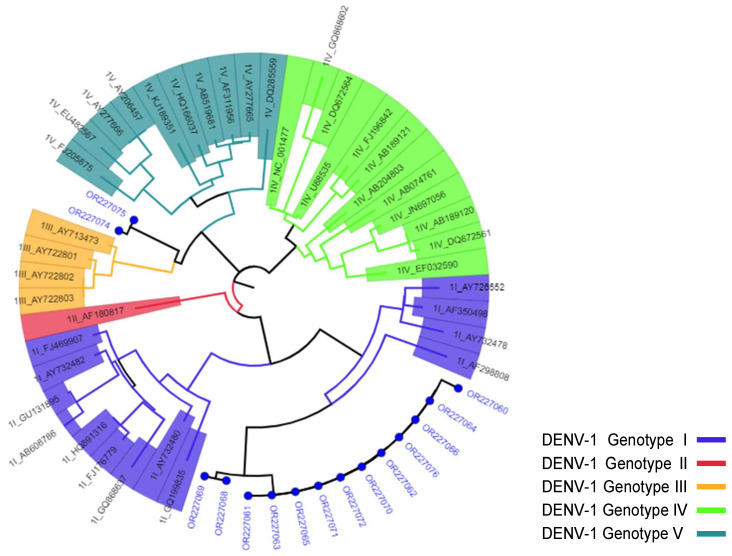
Genotyping of DENV-1 strains using the Dengue Virus Typing Tool. Blue circles represent the strains from this study, while others are reference strains, represented with their GenBank accession numbers and numbers representing the serotype and genotypes included in the analysis.

**Figure 3:**
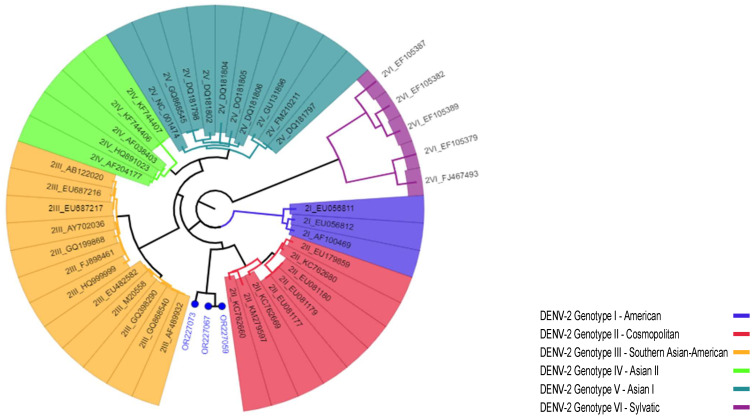
Genotyping of DENV-2 strains using the Dengue Virus Typing Tool. Blue circles represent the strains from this study, while others are reference strains, represented with their GenBank accession numbers and numbers representing the serotype and genotypes included in the analysis.

Regarding the agreement of the two genotyping tools we employed, 13 of the 15 DENV-1 sequences from this study were concordantly assigned Genotype-I by both genotyping tools, while the other two DENV-1 sequences [OR227074 and OR227075], and none of the three DENV-2 strains [OR227059, OR227067, and OR227073] were not assigned to a specific genotype by the Dengue Virus Typing (Figure 2 and Figure 3).

### 3.3. Phylogenetic Analysis

**Figure 4:**
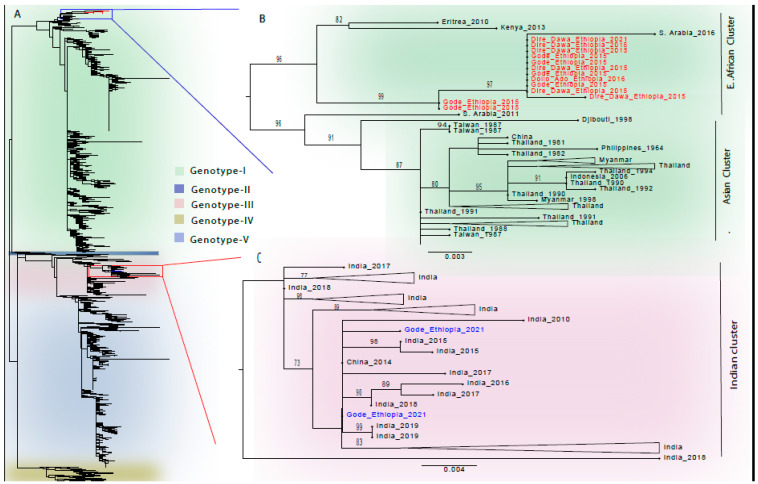
**Phylogenetic analysis of DENV-1 sequences from outbreaks in Eastern and Southeastern Ethiopia.** Evolutionary history was inferred by using the maximum-likelihood phylogenies. (**A**) Phylogenetic tree topology of DENV-1 sequences (N = 2442), classified by genotypes. The Genotype-I and Genotype-III sequences from this study are shown in red and blue branches, respectively. (**B**) Phylogenetic tree of the selected DENV-1 Genotype-I sequences is indicated by the blue rectangle in the light-green-shaded section of the panel (**A**). The sequences from this study are shown in red. (**C**) Phylogenetic tree of the selected DENV-1 Genotype-III sequences is indicated by the red rectangle in the light-pink-shaded section of the panel (**A**). The sequences from this study are shown in blue. Panel (**B**,**C**): sequences in the trees are shown by their country of origin and year of sample collection. The scale bar indicates the genetic distance. Only bootstrap values greater than 70% are shown in the phylogenic trees.

The DENV-1 sequences from this study are clustered into two distinct genotype-specific clusters, as seen in the topology of the DENV-1 tree (Figure 4A and Figure S1A). Eleven of the 13 DENV-1 Genotype-I sequences from this study clustered together in a monophyletic lineage, representing samples from Dire Dawa in 2015 and the Somali Region in 2015 and 2016, as well as one sequence from Dire Dawa in 2021 (Figure 4B and Figure S1B). This lineage also contained a sequence from Saudi Arabia in 2016. The other two DENV-1 Genotype-I sequences from our study formed a related cluster just outside of this lineage (Figure 4B and Figure S1B). The most closely related non-Ethiopian sequences sharing a common ancestor with these DENV-I, Genotype-I sequences are from neighboring countries of Eritrea and Kenya, in 2010 and 2013, respectively. All these sequences were most closely related to the cluster formed by sequences from Southeast Asian countries (Figure 4B). The Ethiopian DENV-1 Genotype-I sequences and the sequences from Kenya, Eritrea, and Saudi Arabia fell into an isolated cluster in the DENV-1 serotype-specific phylogenetic tree, distinctly located in the tree topology (Figure 4A). Two DENV-1 Genotype-III sequences from the 2021 Gode outbreak clustered with sequences almost exclusively from India between 2010 to 2019, with only one sequence from China in 2014 in the cluster (Figure 4C and Figure S1C).

**Figure 5:**
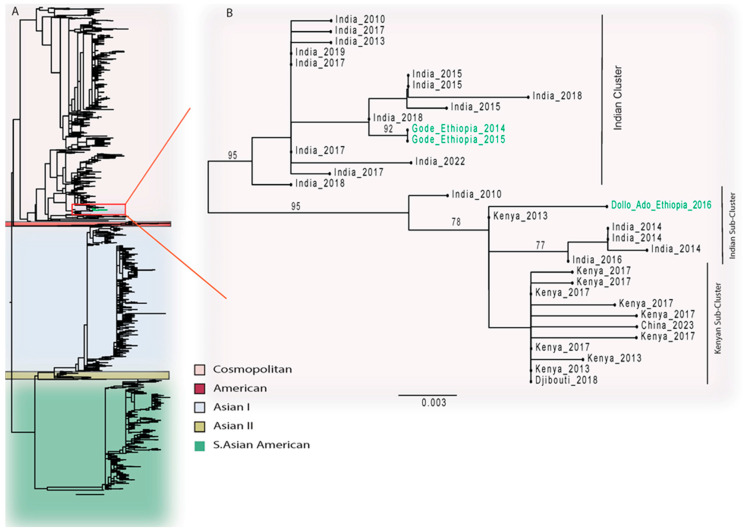
**Phylogenetic analysis of DENV-2 sequences from outbreaks in Eastern and Southeastern Ethiopia.** Evolutionary history was inferred by using the maximum-likelihood phylogenies. (**A**) Phylogenetic tree topology of DENV-2 serotype sequences (N = 1911), classified by genotypes. The sequences from this study are shown in green branches. (**B**) The phylogenetic tree of the selected DENV-2, Cosmopolitan Genotype sequences is indicated by the red rectangle in the light-pink-shaded section of the panel (**A**). Sequences in the tree are shown by their country of origin and year of sample collection. The sequences from this study are shown in green. The scale bar indicates the genetic distance. Only bootstrap values greater than 70% are shown in the phylogenic trees.

The three DENV-2 sequences from the current study belonged to the Cosmopolitan Genotype (Figure 5A and Figure S2A). Two of these, from the 2014 and 2015 outbreaks in Gode, formed a monophyletic lineage and clustered together with sequences exclusively from India in 2015 and 2018, while one associated with the 2016 outbreak in Dollo Ado, clustered with sequences from Kenya and others from India (Figure 5B and Figure S2B).

## 4. Discussion

The present study is the first attempt to serotype, genotype, and phylogenetically analyze the DENV strains responsible for dengue outbreaks in Ethiopia. Our results showed that at least two serotypes, DENV-1 and DENV-2, were responsible for the documented dengue outbreaks, with a predominance of DENV-1 (15 out of the 18 sequences serotyped). This finding is in agreement with a recent systematic review and meta-analysis of dengue outbreaks in Africa, where DENV-1 and DENV-2 were the most commonly identified serotypes in outbreaks that occurred between 1964 and 2020, with a predominance of DENV-1 (4). From our results, it appears that DENV-2 co-circulated along with the predominant DENV-1 during the outbreaks in Gode in 2015 and Dollo Ado in 2016. The co-circulation of different DENV serotypes can be considered a warning sign that a heterologous infection could occur and be a risk factor for severe dengue. This finding also emphasizes the need for the routine monitoring of circulating serotypes and genotypes. The fact that DENV-1 is the dominant serotype identified in the samples collected during the dengue outbreaks included in the current study, characterized as less severe, is in line with some correlational studies where DENV-1 was found to cause milder illness during epidemics [16,22,23].

Our eleven DENV-1 Genotype-I sequences clustered together, though they were collected from different locations: Dire Dawa, Gode and Dollo Ado; and at different years: 2015–2021. This demonstrates that closely related DENV-1 strains circulated in these areas for years, suggesting continued local transmission with minimal genetic divergence, at least with respect to the CprM gene sequenced in this study. These DENV-1 Genotype-I sequences also clustered closely with the sequences from Eritrea and Kenya, suggesting regional transmission among these border-sharing neighboring countries. DENV-1 Genotype-I has been frequently reported by East African countries such as in Djibouti in 1998, Eritrea in 2010, Somalia in 2011, and Kenya in 2013 [24], and in our attempt to genotype all the DENV-1 sequences from the African region available as of 20 March 2024 in the NCBI Virus database (with 72.4% of the sequences from Tanzania, Table S1), we found that the majority of the sequences (79.1%) were assigned Genotype-III (Table S2). However, when sequences from Tanzania were excluded, the genotyping data from East Africa showed the majority (61.5%) of the sequences to be Genotype-I (Table S3). A recent time-scaled phylogenetic analysis of DENV in Africa showed two geographically distinct clusters of DENV-1 in Eastern Africa, with cluster I restricted to the northern countries of Eastern Africa, including Eritrea and Kenya, estimated to be of relatively recent origin [25]. The monophyletic clustering of a sequence from Saudi Arabia with the Ethiopian DENV-1 Genotype-I sequences is also interesting and shows the possible DENV transmissions between the two countries

The detection of the two DENV-1 Genotype-III strains in the outbreak in 2021 in Gode is important and likely a new introduction, as strains that circulated previously and were sequenced in this study were Genotype-I. Despite the circulation of Genotype-III in Eastern Africa (Table S1), no African Genotype-III sequences clustered with the sequences from this study. Interestingly, nearly all of the sequences that clustered with our Genotype-III sequences were from India, collected between 2010 and 2019; one additional sequence was from China (Figure 4C). Therefore, it may be likely that the DENV-1 Genotype-III strains were imported to Ethiopia from India.

Since there was no linking of clinical data with the circulated genotypes in our study, it was not possible to compare the clinical outcomes and severity of infections by the two DENV-1 genotypes.

All three DENV-2 genotypes in our study were found to be the Cosmopolitan Genotype, which is consistent with a report of a phylogenetic study of circulating DENV in Africa [26]. This also complemented finding of the our genotyping effort of DENV-2 sequences from the African region available as of 7 April 2024 in the NCBI Virus Database, where we found 78.8% of the sequences to be the Cosmopolitan Genotype (Tables S4 and S5). The monophyletic nature of the two strains that circulated in the Gode outbreaks in 2014 and 2015 indicates a continued and local transmission of these strains. Interestingly, these sequences cluster with sequences solely from India, and this corroborates with previous reports of circulation of the Indian sub-continent lineage, particularly in East Africa [27]. It was noted that another Cosmopolitan sequence from Dollo Ado (a town close to the Ethiopian–Kenyan border in Southeastern Ethiopia) in 2016 clustered with the sequences from Kenya in 2013. The movement of people across the border between these two countries may be responsible for the importation of the strain to Ethiopia given that the strain circulated earlier in Kenya. Studies to correlate the severity of dengue with dengue serotypes have found that infection with DENV-2 has a higher risk of increased severity [16,28,29]. Even though DENV-2 strains (n = 3) were too few to make a correlation with dengue severity, they circulated during outbreaks in which no death or dengue shock syndrome was reported [30].

It is to be acknowledged that the current study is not without limitations. First, there are limited clinical and meta-data of cases to correlate disease severity with dengue serotypes/genotypes. Second, the number of sequenced DENV strains is low. Even among the 39 serum samples that were collected during the 2014–2016 and 2021 dengue outbreaks in Eastern and Southeastern Ethiopia and were confirmed to harbor DENV RNA by RT-PCR previously, the redetection of the RNA was possible for only 21 of them. Long-term storage of the samples or sub-optimal storage conditions could be implicated for RNA degradation and un-detection in 18 of the samples. In addition, this discrepancy may be due to the differences in sensitivity and specificity of the PCR test used during the previous outbreak confirmation and redetection in our study. Therefore, all the DENV serotypes and genotypes that circulated during the outbreaks may not be fully represented in the findings of our study. Finally, in our study, we sequenced the CprM gene for the serotyping and genotyping of the dengue viruses from this study. The sequencing of other regions of the genome, such as non-structural genes, the 5′ and 3′ untranslated envelope gene or whole-genome sequencing, could have provided more detailed characteristics and epidemiology of the DENV strains that circulated during the outbreaks.

## 5. Conclusions

Our study showed that the 2014–2016 and 2021 dengue outbreaks that occurred in Eastern and Southeastern parts Ethiopia were predominately due to DENV-1 Genotype-I infections. Our finding also showed that both DENV-1 and DENV-2 co-circulated during the 2015 outbreak in Gode and the 2016 outbreak in Dollo Ado. All three DENV-2 serotypes were genotyped as Cosmopolitan. The phylogenetic analyses revealed that the DENV strains that were responsible for the outbreaks are related to the DENV strains circulating in neighboring countries such as Kenya and Eritrea, as well as Asian countries such as India and China, with the possibility of introductions and exportations of DENV to and from Ethiopia. The formation of a monophyletic lineage by the majority of DENV-1 sequences from the strains that circulated in the outbreaks of 2015, 2016, and 2021 in Dire Dawa City and affected areas in the Somali Region also indicated the continued local transmission of dengue viruses after the initial introduction, calling for enhanced efforts for breaking the circulation cycle of dengue and the related vector-borne pathogens in the recurrently affected regions. Both DENV-1 and DENV-2 were found co-circulating during the 2015 outbreak in Gode and the 2016 outbreak in Dollo Ado. As infections with heterologous DENV serotypes can be a risk factor for developing severe dengue, it is important to monitor and detect DENV serotypes and genotypes. There is a strong need to strengthen DENV molecular surveillance capacity at central and regional levels, especially in the areas where recurrent dengue outbreaks occur.

## Figures and Tables

**Table 1:** Result of the serotyping and genotyping of the sequences from this study.

Accession No.	Year of Outbreak	District/Region Affected	Serotype/Genotype *
OR227059	2014	Gode/Somali	DENV-2/Cosmopolitan
OR227060	2015	Dire Dawa City	DENV-1/Genotype-I
OR227061	2015	Dire Dawa City	DENV-1/Genotype-I
OR227062	2015	Dire Dawa City	DENV-1/Genotype-I
OR227063	2015	Dire Dawa City	DENV-1/Genotype-I
OR227064	2015	Dire Dawa City	DENV-1/Genotype-I
OR227065	2015	Gode/Somali	DENV-1/Genotype-I
OR227066	2015	Gode/Somali	DENV-1/Genotype-I
OR227067	2015	Gode/Somali	DENV-2/Cosmopolitan
OR227068	2015	Gode/Somali	DENV-1/Genotype-I
OR227069	2015	Gode/Somali	DENV-1/Genotype-I
OR227070	2015	Gode/Somali	DENV-1/Genotype-I
OR227071	2015	Gode/Somali	DENV-1/Genotype-I
OR227072	2016	Dollo Ado/Somali	DENV-1/Genotype-I
OR227073	2016	Dollo Ado/Somali	DENV-2/Cosmopolitan
OR227074	2021	Gode/Somali	DENV-1/Genotype-III
OR227075	2021	Gode/Somali	DENV-1/Genotype-III
OR227076	2021	Dire Dawa City	DENV-1/Genotype-I

* Analysis performed using the Flavivirus Genotyping Tool.

## Data Availability

The sequence data presented in this study are openly available in the GenBank database, accession numbers OR227059–OR227076.

## Supplementary Materials

The following supporting information can be downloaded at: https://www.mdpi.com/article/10.3390/v16081334/s1, Table S1. Countries contributing > 10 DENV-1 sequences to the NCBI Virus Database of the African Region; Table S2 Genotyping results of DENV-1 sequences from the African Region; Table S3. Genotyping results of the sequences from East African countries, excluding Tanzania; Table S4. Countries contributing > 10 DENV-2 sequences to the NCBI Virus Database of the African Region; Table S5 Genotyping results of DENV-2 sequences from the African Region; Figure S1: Phylogenetic analysis of DENV-1 sequences from outbreaks in Eastern and Southeastern Ethiopia; Figure S2: Phylogenetic analysis of DENV-2 sequences from outbreaks in Eastern and Southeastern Ethiopia.

## References

[1] Green S., Rothman A. (2006). Immunopathological mechanisms in dengue and dengue hemorrhagic fever. Curr. Opin. Infect. Dis..

[2] Holmes E.C., Twiddy S.S. (2003). The origin, emergence and evolutionary genetics of dengue virus. Infect. Genet. Evol..

[3] World Health Organization: WHO Dengue and Severe Dengue. Fact Sheet. March 2023. https://www.who.int/news-room/fact-sheets/detail/dengue-and-severe-dengue.

[4] Eltom K., Enan K., El Hussein A.R.M., Elkhidir I.M. (2021). Dengue Virus Infection in Sub-Saharan Africa Between 2010 and 2020: A Systematic Review and Meta-Analysis. Front. Cell. Infect. Microbiol..

[5] Woyessa A.B., Mengesha M., Kassa W., Kifle E., Wondabeku M., Girmay A., Kebede A., Jima D. (2014). The first acute febrile illness investigation associated with dengue fever in Ethiopia, 2013: A descriptive analysis. Ethiop. J. Health Dev..

[6] Amarasinghe A., Kuritsky J.N., Letson G.W., Margolis H.S. (2011). Dengue Virus Infection in Africa. Emerg. Infect. Dis..

[7] Degife L.H., Worku Y., Belay D., Bekele A., Hailemariam Z. (2019). Factors associated with dengue fever outbreak in Dire Dawa administration city, October, 2015, Ethiopia-Case control study. BMC Public Health.

[8] Mesfin Z., Ali A., Abagero A., Asefa Z. (2022). Dengue Fever Outbreak Investigation in Werder Town, Dollo Zone, Somali Region, Ethiopia. Infect. Drug Resist..

[9] Mekuriaw W., Kinde S., Kindu B., Mulualem Y., Hailu G., Gebresilassie A., Sisay C., Bekele F., Amare H., Wossen M. (2022). Epidemiological, Entomological, and Climatological Investigation of the 2019 Dengue Fever Outbreak in Gewane District, Afar Region, North-East Ethiopia. Insects.

[10] Gutu M.A., Bekele A., Seid Y., Mohammed Y., Gemechu F., Woyessa A.B., Tayachew A., Dugasa Y., Gizachew L., Idosa M. (2021). Another dengue fever outbreak in Eastern Ethiopia—An emerging public health threat. PLoS Negl. Trop. Dis..

[11] Gemechu D.S., Worku Y., Alemu A., Edea Z.A., Feyisa Y.D., Watere S.H., Belay D., Hagos A., Woyessa A.B. Fourth Dengue Fever Outbreak Investigation in Ethiopia: A Case Control Study. July 2015. https://www.researchsquare.com/article/rs-4729/v1.

[12] Soo K.M., Khalid B., Ching S.M., Chee H.Y. (2016). Meta-analysis of dengue severity during infection by different dengue virus serotypes in primary and secondary infections. PLoS ONE.

[13] Anoop M., Issac A., Mathew T., Philip S., Kareem N.A. (2010). Genetic characterization of dengue virus serotypes causing concurrent infection in an outbreak in Ernakulam, Kerala, South India. Indian J. Exp. Biol..

[14] Caroline A., Bona D., Twerdochlib A.L. (2012). Genetic diversity of dengue virus serotypes 1 and 2 in the State of Paraná, Brazil, based on a fragment of the capsid/premembrane junction region. Rev. Soc. Bras. Med. Trop..

[15] Luo Z., Hu B., Zhang F., Lin X., Xie X., Pan K., Li H.Y., Ren R.W., Zhao W.Z. (2017). Laboratory and Molecular Characterization of Dengue Viruses in a 2014 Outbreak in Guangfo Region, Southern China. PLoS Negl. Trop. Dis..

[16] Falk K.I., Alm E., Lesko B., Lindegren G., Ahlm C., So S., Lagerqvist N. (2014). Universal Single-Probe RT-PCR Assay for Diagnosis of Dengue Virus Infections. PLoS Negl. Trop. Dis..

[17] Lanciotti R.S., Calisher C.H., Gubler D.J., Chang G.J., Vorndam A.V. (1992). Rapid detection and typing of dengue viruses from clinical samples by using reverse transcriptase-polymerase chain reaction. J. Clin. Microbiol..

[18] Fonseca V., Libin P.J.K., Theys K., Faria N.R., Nunes M.R.T., Restovic M.I., Freire M., Giovanetti M., Cuypers L., Nowé A. (2019). A computational method for the identification of dengue, zika and chikungunya virus species and genotypes. PLoS Negl. Trop. Dis..

[19] Vilsker M., Moosa Y., Nooij S., Fonseca V., Ghysens Y., Dumon K., Pauwels R., Alcantara L.C., Vanden Eynden E., Vandamme A.M. (2019). Genome Detective: An automated system for virus identification from high-throughput sequencing data. Bioinformatics.

[20] Nguyen L.T., Schmidt H.A., Von Haeseler A., Minh B.Q. (2015). IQ-TREE: A fast and effective stochastic algorithm for estimating maximum-likelihood phylogenies. Mol. Biol. Evol..

[21] Rambaut A. (2010). FigTree. Institute of Evolutionary Biology, University of Edinburgh, Edinburgh. http://tree.bio.ed.ac.uk/software/figtree/.

[22] Kalayanarooj S., Nimmannitya S. (2000). Clinical and laboratory presentations of dengue patients with different serotypes. WHO Regional Office for South-East Asia. Dengue Bull..

[23] Corwin A.L., Larasati R.P., Bangs M.J., Wuryadi S., Arjoso S., Sukri N., Listyaningsih E., Hartati S., Namursa R., Anwar Z. (2001). Epidemic dengue transmission in southern Sumatra, Indonesia. Trans. R. Soc. Trop. Med. Hyg..

[24] Andre G., Lamballerie XDe Leparc-goffart I., Grard G. (2023). Molecular characterization of dengue virus serotype 1 infections in French travelers from Africa between 2013 and 2019. Front. Virol..

[25] Alfsnes K., Eldholm V., Gaunt M.W., De Lamballerie X., Gould E.A., Pettersson J.H. (2021). Tracing and tracking the emergence, epidemiology and dispersal of dengue virus to Africa during the 20th century. One Health.

[26] Ayolabi C.I., Olusola B.A., Ibemgbo S.A., Okonkwo G.O. (2019). Detection of Dengue viruses among febrile patients in Lagos Nigeria and phylogenetics of circulating Dengue serotypes in Africa. Infect. Genet. Evol..

[27] Yenamandra S.P., Koo C., Chiang S., Shi H., Lim J., Yeo Z.Y., Ng L.C., Hapuarachchi H.C. (2021). Evolution, heterogeneity and global dispersal of cosmopolitan genotype of Dengue virus type 2. Sci. Rep..

[28] Vicente C.R., Herbinger K.H., Fröschl G., Romano C.M., Cabidelle A.d.S.A., Junior C.C. (2016). Serotype influences on dengue severity: A cross-sectional study on 485 confirmed dengue cases in Vitória, Brazil. BMC Infect. Dis..

[29] Gupta A., Rijhwani P., Pahadia M.R., Kalia A., Choudhary S., Bansal D.P., Gupta D., Agarwal P., Jat R.K. (2021). Prevalence of Dengue Serotypes and Its Correlation With the Laboratory Profile at a Tertiary Care Hospital in Northwestern India. Cureus.

[30] Ahmed Y.M., Salah A.A. (2016). Epidemiology of Dengue Fever in Ethiopian Somali Region: Retrospective Health Facility Based Study. Cent. Afr. J. Public Health.

