# Supplementary material for: Predominance of Dengue Virus Serotype-1/Genotype-I in Eastern and Southeastern Ethiopia
Source: Viruses. 2024 Aug 21;16(8):1334. doi: 10.3390/v16081334 (PMC11359325; doi:10.3390/v16081334)
Supplement: Supplementary file 1 [file viruses-16-01334-s001.zip › Tables S1-S5.pdf]

Table S1. Countries contributing > 10 DENV-1 sequences to the NCBI Virus Database of the African Region*

Country	Number of Sequences	% Contribution
**Tanzania**	346	72.4
**Reunion**	19	4.0
**Ethiopia**	15	3.1
**DRC**	13	2.7
**Angola**	12	2.5
**Total**	**405**	**84.7**

*Based on number of sequences in the NCBI Virus Database of the African Region as of March 20, 2024

Table S2. Genotyping results of DENV-1 sequences from the African Region *

Genotype†	Number	Proportion
Genotype-III	378	79.1
Genotype-I	34	7.1
Genotype-IV	11	2.3
Genotype-V	10	2.1
Genotype-Sylvatic	5	1.0
Unassigned	36	7.5
Blank	4	0.8
Total	478	100.0

*Analysis is based on sequences in the NCBI Virus Database of the African Region as of March 20, 2024
† Genotyping done using the Flavivirus Genotyping Tool

Table S3. Genotyping results of sequences from East Africa countries, excluding Tanzania

Genotype†	Number	%
Genotype-III	5	19.2
Genotype-I	16	61.5
Genotype-Sylvatic	5	19.2
Total	26	100.0

*Analysis is based on sequences in the NCBI Virus Database of the African Region as of March 20, 2024
† Genotyping done using the Flavivirus Genotyping Tool

Table S4. Countries contributing > 10 DENV-2 sequences to the NCBI Virus Database of the African Region*

Country	Number of Sequences	% Contribution
Kenya	58	22.0
Angola	26	9.8
Burkina Faso	33	12.5
Senegal	25	9.5
Tanzania	16	6.1
Nigeria	19	7.2
Mauritania	11	4.2
Ivory Coast	12	4.5
Total	200	75.8

*Based on number of sequences in the NCBI Virus Database of the African Region as of April 7, 2024

Table S5. Genotyping Results DENV-2 sequences from the African Region*

Genotype†	Number	%
Genotype-Cosmopolitan	208	78.8
Genotype-Asian I	12	4.5
Genotype-Asian II	10	3.8
Genotype-Southern Asian American	4	1.5
Genotype-Sylvatic	23	8.7
Blank	7	2.7
Total	264	100.0

*Analysis is based on sequences in the NCBI Virus Database of the African Region as of April 7, 2024
† Genotyping done using the Flavivirus Genotyping Tool
