# Supplementary figures and images for: Predominance of Dengue Virus Serotype-1/Genotype-I in Eastern and Southeastern Ethiopia
Source: Viruses. 2024 Aug 21;16(8):1334. doi: 10.3390/v16081334 (PMC11359325; doi:10.3390/v16081334)

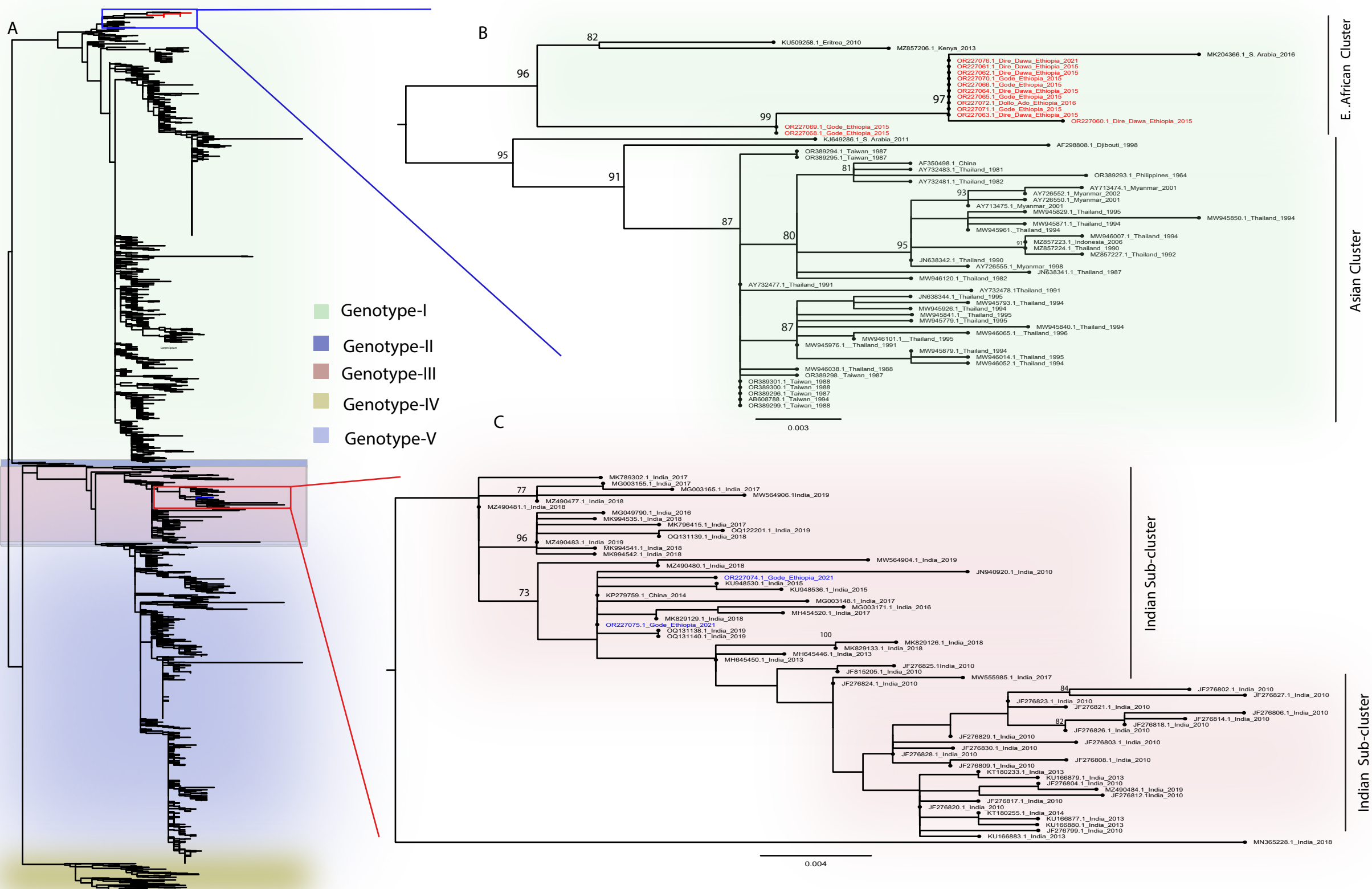

Supplement: Supplementary file 1 [file viruses-16-01334-s001.zip › Figure S1.pdf.pdf]

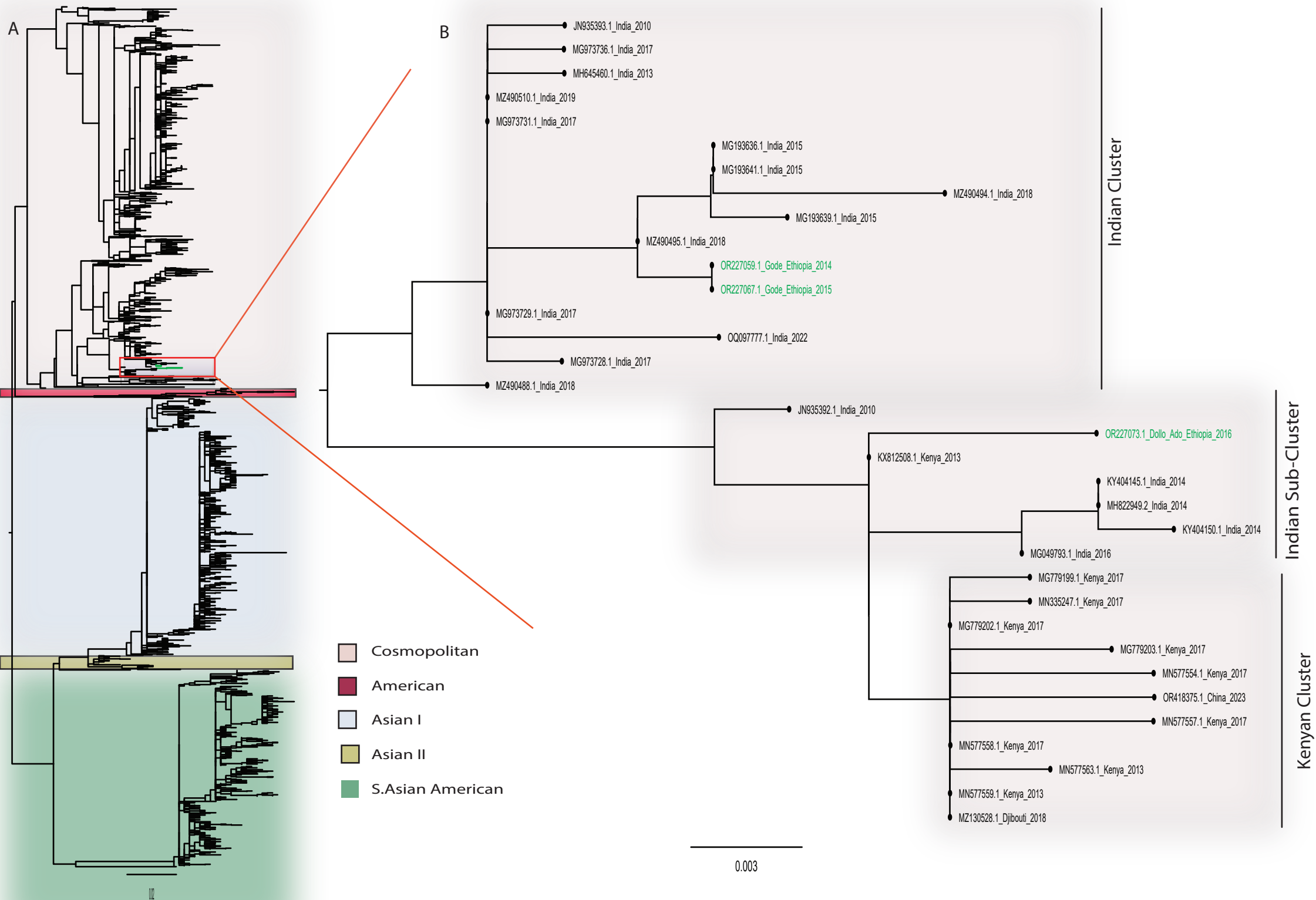

Supplement: Supplementary file 1 [file viruses-16-01334-s001.zip › Figure S2.pdf.pdf]
